# Predictive and Prognostic Value of sPRR in Patients with Primary Epithelial Ovarian Cancer

**DOI:** 10.1155/2016/6845213

**Published:** 2016-08-31

**Authors:** Katrin Kreienbring, Annika Franz, Rolf Richter, Duska Dragun, Harald Heidecke, Ralf Dechend, Dominik N. Muller, Jalid Sehouli, Elena I. Braicu

**Affiliations:** ^1^Department of Gynecology, Campus Virchow Klinikum, Charité Universitätsmedizin Berlin, Berlin, Germany; ^2^Department of Nephrology and Intensive Care Medicine, Charité Universtätsmedizin Berlin, Berlin, Germany; ^3^CellTrend, Luckenwalde, Germany; ^4^Experimental and Clinical Research Center, Berlin, Germany

## Abstract

*Aim*. The purpose of the present study was to analyze the predictive and prognostic role of soluble (pro)renin receptor (sPRR) as a biomarker for clinicopathological outcome in patients with primary epithelial ovarian cancer (EOC). As part of the renin-angiotensin system (RAS) whose activity is known to increase in ovarian cancer patients, the relation of sPRR and ovarian cancer should be further investigated.* Patients and Methods*. In this study 197 patients with primary EOC in our institution from 2000 to 2011 were included. sPRR was determined by enzyme-linked immunosorbent assay (ELISA) in preoperative taken blood sera. Associations with clinicopathological outcome were analyzed and serum levels of sPRR in patients have been compared to those in healthy specimen. Kaplan-Meier and logistic/Cox regression assessed the impact of the markers on progression-free survival (PFS) and overall survival (OS).* Results*. There have been no correlations proved of sPRR levels with neither clinicopathological factors nor prognostic data. Also the distribution of sPRR in patients and controls was normal.* Conclusion*. sPRR seems to have no predictive, prognostic, or diagnostic value in EOC. As several factors of the RAS which might indicate cancer events have been shown, sPRR seems not to be affected.

## 1. Background

Epithelial ovarian cancer (EOC) is the leading cause of death for gynaecological cancers. Its poor prognosis is contingent on various factors: (a) nearly 70% of the concerned women have advanced FIGO stages with extrapelvic metastasis at diagnosis [1], (b) more than 20% are resistant to chemotherapy, (c) furthermore EOC's molecular complexity makes targeted chemotherapy and potent diagnostic marker difficult to establish, and (d) more than 60% of the patients will relapse [2]. Currently standard of care is debulking surgery followed by platinum-taxane chemotherapy. After surgery the largest diameter of the residual tumour mass is recorded and acts with FIGO stage at diagnosis as the strongest prognostic factor [3]. Moreover ascites before surgery [4] and the temporal context of debulking surgery and chemotherapy [5] have a strong clinical prognostic value as well. The ROVAR (Risk Of Ovarian Cancer Relapse) score categorises EOC patients into three risk groups by determining preoperative CA-125, FIGO stage, histological grading, and presence of residual disease at posttreatment [2] and accomplishes a sensitivity and specificity for relapse of 94% and 61%.

With the determination of EOC's tumorigenesis and its certain histopathologies and molecular subclassifications the development of specific therapies for the different types of EOC becomes achievable. Predictive or prognostic associations with such subgroups might occur as well.

Therefore on the one hand powerful diagnostic markers for earlier diagnosis and on the other hand predictive and prognostic markers to dedicate suitable targeted therapies to the patients are needed. They can be used to identify the most benefitting therapy management for those women. Therefore random tumour markers and markers that have been found upregulated in the environment of EOC are in the focus of research. In this study we determined the soluble form of (pro)renin receptor (PRR).

*The (pro)renin receptor* (PRR) has four main functions in the human body. Initially the receptor was described by Ludwig et al. in 1998 as a component of V-ATPase and called ATP6M8-9 [6]. Later it was discovered by Nguyen et al. in 2002 that linking renin or prorenin to this receptor (a) starts the cascade of tissue renin-angiotensin system (RAS) with the conversion of angiotensinogen into angiotensin I and (b) also initiates intracellular mitogen-activated protein kinase (MAPK) pathways [7]. In recent studies PRR has been revealed to act as an adaptor protein between V-ATPase and LRP6, which are parts of the Wnt receptor complex [8]. Such Wnt cascades are known to affect oncogenesis [9] and cardiorenal end-organ damage [10].

As there are studies proving that the activity of the RAS is increased in ovarian cancer patients, we decided to investigate also the cascade-starting receptor PRR. Such increased activity is characterised by, for example, angiotensin II type 1 receptor (AGTR1), which has a prognostic role in EOC [11], or angiotensin converting enzyme (ACE) which is upregulated in cancer patients [12]. The presence of a soluble form (sPRR) makes the receptor more likely to become an easy quantifiable marker. It is proved that blocking the AGTR1 with candesartan in vitro and in mice reduces peritoneal carcinomatosis, decreases ascitic VEGF concentration, and prevents angiogenesis [13]. Therefore a possible similar impact of sPRR should be investigated.

## 2. Materials and Methods

### 2.1. Patients

In this study 197 patients with primary EOC were included. The serum samples have been obtained before cytoreductive surgery between 2000 and 2011. They were collected and the data of the patients was regularly updated by the Tumor Bank of Ovarian Cancer (http://www.toc-network.de/). TOC is a multicentre project at the Department of Gynaecology at Campus Virchow Clinics, Berlin, which started in 2000 and is still running. The clinics have an emphasis on multimodal therapy of ovarian cancer for more than 15 years. The follow-up of all patients in this study ended 2013 and has a mean of 44.8 months (range 3–114) only ending with the death of a patient.

The median age of the patients at date of diagnosis was 60 years ranging from 28 to 92 years. 181 patients (91.9%) had already advanced tumour disease (FIGO III/IV) at presentation. All patients were treated with debulking surgery and 170 patients (86.3%) got chemotherapy over 6.2 cycles on average (ranged 1–16). Taxol and carboplatin were given to 154 (78.2%) women, 11 (5.6%) were treated with other platinum containing combinations, and 5 patients (2.5%) received immunotherapy.

For comparison 132 specimens from healthy asymptomatic women were collected at the University Medical Center Göttingen. All serum specimens were processed using the same protocol. Their median age was 57.5 years (range 42–83).

The serum levels of sPRR have been assayed by CellTrend GmbH (http://www.celltrend.de/) with ELISA.

### 2.2. ELISA

To determine sPRR CellTrend uses a direct sandwich ELISA. The specific antibody is precoated onto a microplate. The samples and standards are pipetted into the wells and incubated for 120 minutes. Following this, any sPRR present is bound. After washing a biotin-labelled antibody is added and incubated again for 60 minutes. Thereafter another wash follows and enzyme-linked streptavidin subjoins. A substrate solution is pipetted to the wells after 60 mins of incubation and washing. In 30 minutes of incubation a colourful solution establishes. The absorption is measured at 450 nm with an ELISA reader and behaves proportionally to the sPRR concentration. The sPRR levels are labelled in U per millilitre.

### 2.3. Statistical Methods

For the statistical evaluations IBM SPSS Statistics ver. 22 (SPSS Inc., Chicago, IL, USA) was used. Spearman's rho, Kruskal-Wallis test, and Mann-Whitney *U* test were applied to determine the association between sPRR levels and clinicopathological factors as well as between patients and healthy controls. The *α*-level was assumed as <0.05. Kaplan-Meier analysis provided quartile estimates. The survival distributions have been compared with log-rank (Mantel-Cox). Any clinically or histologically confirmed cancer recurrence was defined as an event for the calculation of progression-free survival (PFS). The overall survival (OS) is defined as the interval between date of diagnosis and the death of the patients.

The clinical and pathological factors were mass of ascites, FIGO classification, age at diagnosis, residual tumour mass after surgery, histological WHO grading, histology, response to platinum-based therapy, and PFS and OS in patients with primary EOC.

Ethical approval was obtained from the Ethical Committee, Charité Medical University, Berlin (number 207/2003), University Medical Center Göttingen (no. EK 22/2/04). Written informed consent was provided by the patients before enrolment and serum sample collection.

## 3. Results

In this study we investigated serum samples of 197 patients with primary EOC. The median age of the group was 60 years, ranging from 28 to 92. 91.9% of the patients had advanced FIGO stage III or IV. The distribution of histology, histological grading, volume of ascites before surgery, and existence of peritoneal carcinomatosis is shown in Table 1.

All included patients had cytoreductive surgery after taking the serum samples, whereas 2 patients (1.02%) got neoadjuvant chemotherapy and 35 patients (17.8%) came for completing surgery next to 2 patients (1.02%) with interval surgery. There was no residual tumour mass after surgery in 103 women (52.3%); on the other hand in 22 (11.2%) cases the biggest diameter of nonresectable tumour was more than 2 cm. Eight patients (4.1%) died from post-op complications and 128 patients (65%) died by now in total.

171 patients were treated with chemotherapy. 116 (58.9%) of the patients reacted sensitively to platinum which was defined according to Gyneacologic Cancer InterGroup (GCIG) criteria as no relapse within six months after platinum-based chemotherapy.

### 3.1. sPRR Expression in Ovarian Cancer Patients versus Healthy Controls

**Figure 1:**
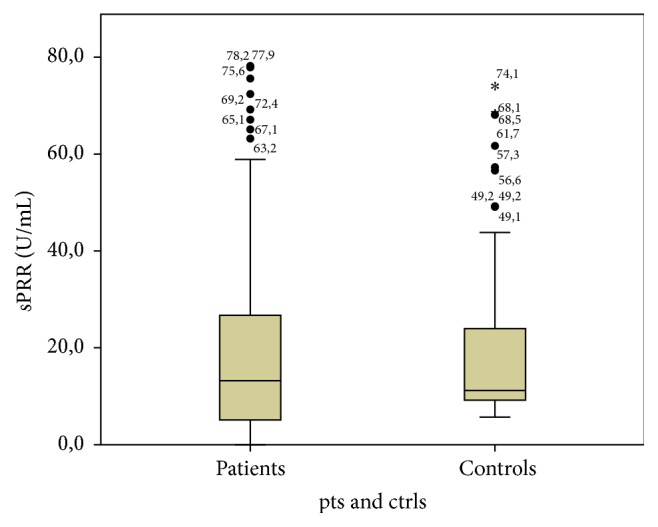
sPRR levels in patients and controls.

The serum levels of sPRR in EOC patients and control group are shown in Table 2. The values did not correlate significantly with the presence of cancer.  Figure 1 illustrates this relation.

### 3.2. Correlation of sPRR Expression with Clinical, Prognostic, and Histological Factors in Ovarian Cancer Patients

The *p* values of the correlations with the clinicopathological factors are shown in Table 3. There are no significant correlations with the sPRR levels.

### 3.3. Impact of sPRR Expression on Survival

The median PFS of the whole group was 14 months ranging from 0 to 114 months. The time of PFS has been defined as period between surgery and occurrence of relapse. Furthermore the whole group had a median OS of 40 months ranging from 0.5 to 114 months. OS-time was defined as the period between diagnosis and death. One-year PFS rate was 61%, 2-year PFS rate was 30%, and after 5 years there have been 6% of the patients without progression. Next to this 1-year OS rate was 79%, 2 year OS rate was 60%, and after 5 years it was 17%. After 8 years 3 patients (1.5%) remained without progression and 6 patients (3%) have not died.  Table 4 shows the distribution of sPRR levels, mean and medians for OS/PFS, and numbers of deaths/progressions.

**Figure 2:**
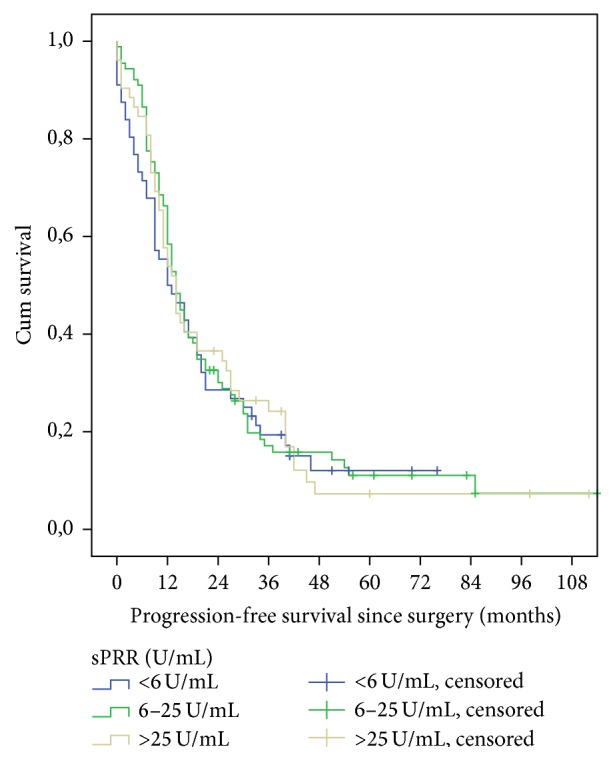
Progression-free survival curves for sPRR-level subgroups (*p* = 0.651).

**Figure 3:**
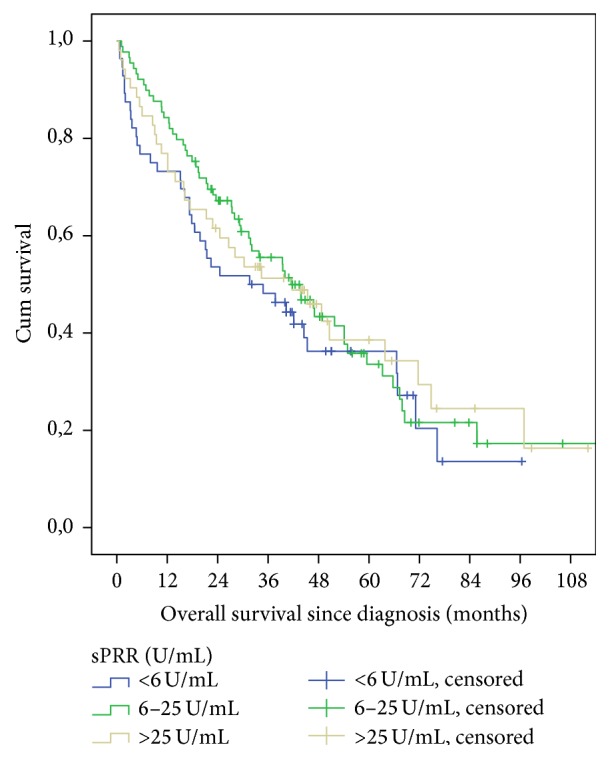
Overall survival curves for sPRR-level subgroups (*p* = 0.395).

Log-rank tests showed no significant relation of OS or PFS with sPRR levels (Table 5, Figures 2 and 3). Likewise there was no correlation in pairwise comparison for the current quartiles.

## 4. Discussion

This study has been designed to analyze a possible biomarker in EOC patients, sPRR, regarding its correlation with several clinical and pathological factors as well as progression-free survival (PFS) and overall survival (OS).

There are no studies to date that have reported on the clinical significance of sPRR and its relationship with survival in ovarian cancer patients. But there might be coherence as sPRR is an important factor in the renin-angiotensin system (RAS), Wnt-cascade, and activation of mitogen-activated protein kinase (MAPK) which all have been shown to be upregulated in ovarian cancer.

### 4.1. Renin-Angiotensin System and Tumorigenesis

Linking prorenin with PRR leads to transformation of angiotensinogen to angiotensin I which can start the RAS [14]. It is known that RAS may be activated in human uterine endometrium, ovary, and placenta both under physiological situations and in malignancies [15–17]. An overview of Deshayes and Nahmias showed that the RAS may be part of angiogenesis, cellular proliferation and apoptosis [18]. In ovarian cancer patients upregulated angiotensin I receptor (AT1R) is known to increase with the tumour invasiveness [13]. Pupilli et al. showed that the activation of AT1R stimulates vascular endothelial growth factor (VEGF) and may lead to angiogenesis in malignant diseases [19]. While angiotensin I seems to upregulate apoptosis and block angiogenesis and proliferation, angiotensin II antagonizes theses effects [20]. A disruptive factor on this balance of the RAS may increase tumorigenesis through neovascularisation, growth, and metastasis [21]. Lever et al. showed that ACE inhibitors may protect against cancer, since the relative risk for female cancers was 0.37 (0.12–0.87) compared with patients receiving other hypertensive drugs in a follow-up of 3 years [22].

Ino et al. investigated 67 ovarian cancer tissues immunohistochemically for AT1R, which was expressed in 85% of the cases. Patients with positive tissues for AT1R had significantly poorer outcome (*p* = 0.041) than those with negative staining. AT1R expression also correlated with increased VEGF [11]. In a study of Suganuma et al. AT1R was immunohistochemically analyzed in 99 ovarian cancer tissues. In this study VEGF was also significantly higher and resulted in enhanced invasiveness when AT1R was expressed. Using candesartan in transplanted mice reduced peritoneal dissemination, decreased ascitic VEGF concentration, and suppressed tumour angiogenesis [13].

Regarding angiotensin converting enzyme (ACE) Beyazit et al. measured serum ACE levels in 41 patients and 19 controls. ACE levels were significantly upregulated in patients with ovarian cancer. There was no correlation recognizable for FIGO stages or pathologic subtypes [12].

### 4.2. Wnt/*β*-Catenin Cascade and Progression-Free Survival in Ovarian Cancer

Next to the transformation of angiotensinogen to angiotensin I, PRR is also capable of starting an intracellular cascade beginning with the activation of promyelocytic leukemia zinc finger (PLZF) protein and ending with the production of Wnt target genes. The Wnt-cascade is a key mediator of cell-cell communication and well known to promote cell proliferation, differentiation, migration, and tissue homeostasis [23].

Dai et al. showed evidence for upregulated genes of the Wnt-cascade in ovarian cancer patients by differential methylation hybridisation of 120 ovarian cancer tumours. They identified two groups of gen loci deferring in their progression-free survival (HR = 2.09; 95% CI (1.39, 3.15)) and a significant correlation with pathologic factors in ovarian cancer patients [24].

### 4.3. Increased Mitogen-Activated Protein Kinase (MAPK) in Drug Resistant Ovarian Cancer

Binding of prorenin receptor also starts the extracellular signal-regulated kinase 1/2 (Erk1/2) by activating MAPK. Erk1/2 is known to increase cell proliferation and upregulate profibrotic factors through the transforming growth factor-*β*1 (TGF-*β*1) [25, 26].

A study of Xie et al. investigates the influence of MAPK in 20 ovarian cancer tissues on Cisplatin sensitivity. The immunohistochemical investigation of those tissues showed significantly higher levels of MAPK in the drug resistant tumours [27].

### 4.4. Limitations

As sPRR is part of the RAS, hypertension might be a confounding factor. Nguyen et al. showed that prorenin receptor levels in high blood pressure patients are not deviating to those of healthy controls [28]. But ACE inhibitors seem to have an impact on sPRR levels. Patients that were treated with RAS blocking medicaments had ≈12% higher sPRR levels than those with other therapies. As ACE inhibitors are first choice in patients with cardiac or kidney diseases and such diseases are known to correlate with increased sPRR levels as well, the confounding factor is not distinct yet.

It is known that a high amount of PRR is located in tissue depending on its perfusion. This may implicate that serum levels are not able to show increasing levels as good as tissue analysis.

### 4.5. Further Investigations

Currently there are no studies analyzing the influence of PRR on RAS, Wnt signalling, or MAPK activation in ovarian cancer. As we analyzed the soluble form in the serum of the patients further investigations may be, for example, immunohistochemical methods or Western Blot to demonstrate PRR directly in ovarian cancer tissue. Prospectively a potential predictive or prognostic marker that can be analyzed after surgery to evaluate best individual therapies is needed for ovarian cancer.

## 5. Conclusion

In this study no predictive, prognostic, or diagnostic values for sPRR in the serum of ovarian cancer patients were shown. There was no prognostic value in the survival analyses. Further studies that analyze the receptor in the ovarian cancer tissue are needed to make out which role PRR plays in the oncogenesis of ovarian cancer.

## Figures and Tables

**Table 1:** Patient's clinical and pathologic characteristics.

*Age in years at diagnosis*	Median 60 (range 28–92)	
*Follow-up period in months*	Mean 44.5 (range 1–114)	

Parameters	*N*	%
	197 patients	

*Histology*		
Serous	183	92.9
Endometrioid	1	0.5
Clear cell tumour	1	0.5
Mixed	4	2.0
Others	8	4.1
*FIGO*		
I	5	2.5
II	11	5.6
III	135	68.5
IV	46	23.4
*Histological grading*		
I	8	4.1
II	49	24.9
III	140	71.0
*Volume of ascites*		
No ascites	47	23.9
<500 mL	77	39.1
>500 mL	73	37.0
*Peritoneal carcinomatosis*		
Present	174	88.3
*Residual tumour mass*		
No residual tumour mass	103	52.3
<0,5 cm	34	17.3
<1 cm	30	15.2
1-2 cm	6	3.0
>2 cm	22	11.2
No tumour	2	1.0
*Response to platinum-based chemotherapy*		
Platinum sensitive	116	58.9
Platinum resistant	55	27.9
No platinum chemo	26	13.2

**Table 2:** Mean and median of serum levels and *p* value of sPRR.

	Mean	Median	Mean	Median	*p* value (Mann-Whitney *U* test)
	In patients (*N* = 197)		In controls (*N* = 200)		
sPRR in U/mL	24.57 (range 0.0–318)	13.2	29.122 (range 5.7–282.8)	11.1	0.119

**Table 3:** *p* value and Spearman's rank for the clinicopathological parameters and sPRR.

Parameters & sPRR	*p* value
Ascites	0.298
FIGO classification	0.066
Age at diagnosis	0.069
Residual tumour mass after surgery	0.224
Grading	0.531
Histology	0.316
Platinum response	0.194

**Table 4:** Mean and median for overall survival (OS) and progression-free survival (PFS) in sPRR groups.

sPRR in U/mL	*N* of patients/%	Mean/medians for OS in months	*N* of deaths (censored)	Mean/medians for PFS in months	*N* of progressions (censored)
<6	56/28.4	39.9/31.6	38 (18)	29.7/12	48 (8)
6–25	89/45.2	49.6/41.5	57 (32)	34.8/14	77 (12)
>25	52/26.4	48.8/41.7	33 (19)	33.8/14	46 (6)

Overall	197/100	47.2/40	128 (69)	35.0/14	171 (26)

**Table 5:** Significance in equality log-rank tests (Mantel-Cox) of sPRR with PFS and OS.

Log-rank (*p*)	Progression-free survival	Overall survival
sPRR	0.651	0.395
